# Frequency and Management of Adverse Drug Reactions Among Drug-Resistant Tuberculosis Patients: Analysis From a Prospective Study

**DOI:** 10.3389/fphar.2022.883483

**Published:** 2022-06-02

**Authors:** Asif Massud, Syed Azhar Syed Sulaiman, Nafees Ahmad, Muhammad Shafqat, Long Chiau Ming, Amer Hayat Khan

**Affiliations:** ^1^ Discipline of Clinical Pharmacy, School of Pharmaceutical Sciences, Universiti Sains Malaysia, George Town, Malaysia; ^2^ Faculty of Pharmaceutical Sciences, Government College University, Faisalabad, Pakistan; ^3^ Faculty of Pharmacy, University of Balochistan, Quetta, Pakistan; ^4^ Programmatic Management of Drug-Resistant Tuberculosis (PMDT) Unit, Nishtar Medical University Hospital, Multan, Pakistan; ^5^ Pengiran Anak Puteri Rashidah Sa'adatul Bolkiah (PAPRSB), Institute of Health Sciences, Universiti Brunei Darussalam, Gadong, Brunei

**Keywords:** adverse drug reactions, frequency, management, drug-resistance tuberculosis, Pakistan

## Abstract

Drug-resistant tuberculosis (DR-TB) management is often linked with a higher rate of adverse drug reactions (ADRs) needing effective and timely management of these ADRs, which, if left untreated, may result in a higher rate of loss to follow-up of drug-resistant patients.

**Study objective:** The study was aimed at prospectively identifying the nature, frequency, suspected drugs, and management approaches for ADRs along with risk factors of ADRs occurrence among DR-TB patients at Nishtar Medical University, Hospital, Multan, Pakistan.

**Materials and Methods:** The prospective study included all the DR-TB patients enrolled for treatment from January 2016 to May 2017 at the study site**.** Patients were evaluated for the treatment-induced ADRs as per standard criteria of the National Tuberculosis Program, Pakistan. Multivariate logistic regression was used to assess the independent variables associated with the occurrence of ADRs.

**Results:** Out of 271 DR-TB patients included in the final analysis, it was observed that 55 patients (20.3%) experienced at least three ADRs. A total of 50 (18.5%) patients experienced zero adverse effects, while 15 (5.5%), 33 (12.2%), and 53 (19.6%) patients experienced one, two, and four ADRs, respectively. Gastrointestinal disturbances (66.7%), nervous system disorders (59.4%), and electrolyte disturbances (55.7%) remained the highest reported ADRs during therapy, followed by arthralgia (49.1%), ototoxicity (24%), pruritic reactions/rash (12.9%), dyspnoea (12.5%), and tinnitus (8.8%). Pulmonary cavitation at the baseline visit (*p*-value 0.001, OR 3.419; 95% CI (1.694–6.902) was significantly associated with the occurrence of ADRs among DR-TB patients.

**Conclusion:** The frequency of ADRs was high among the study cohort; however, these were managed effectively. Patients with recognized risk factors for ADRs occurrence need continuous clinical management efforts.

## Introduction

Tuberculosis (TB), an infectious malaise, has been considered the 13th leading cause of mortality and the second leading infectious killer after COVID-19 around the globe (Cohen, 2021; WHO, 2021). TB is a curable and preventable disease, but the inability to diagnose, delayed diagnosis, malpractices, and irrational use of anti-TB drugs lead to the emergence of drug-resistant tuberculosis (DR-TB) (Pritchard et al., 2003; WHO, 2015). According to the WHO global tuberculosis report in 2021, based on the estimated incidence of TB and DR-TB cases per year, Pakistan stands fifth in terms of drug-susceptible tuberculosis (DS-TB) and fourth in terms of DR-TB (WHO, 2020; WHO, 2021). The emergence of resistant strains of *Mycobacterium tuberculosis* (the causative pathogen for TB) against isoniazid and rifampicin is the main cause of DR-TB over the past decade (Günther, 2014). DS-TB can be effectively treated with first-line anti-TB drugs (FLDs), including isoniazid, rifampicin, ethambutol, and pyrazinamide, while DR-TB treatment requires a broader combination of second-line anti-TB drugs (SLDs), which include fluoroquinolones (levofloxacin and moxifloxacin) and aminoglycosides (amikacin, kanamycin, and capreomycin). Other core SLDs include ethionamide, prothionamide, cycloserine, linezolid, and clofazimine. Add-on drugs include bedaquiline and delamanid. FLDs which retain sensitivity against TB microbe may also be included in the therapeutic regimen (Falzon et al., 2017).

Compliance with treatment guidelines and good microbial diagnosis are considered the main factors for successful treatment outcomes among DR-TB patients. Anti-TB SLDs are not only less effective, more noxious, and costly as compared to FLDs (Basit et al., 2014) but are also linked to more frequent and incurable ADRs. An ADR is defined as “a response to a drug which is noxious and unintended, which occurs at doses normally used in humans for prophylaxis, diagnosis, or therapy of disease, or modification of physiological functions” (WHO, 1972). The nature and frequency of ADRs vary among patients when treated with anti-TB drugs which may result in morbidity and mortality if not detected earlier during treatment (Forget and Menzies, 2006; Gülbay et al., 2006; Tan et al., 2007).

Most of the ADRs that occur are minor and mild in nature and can be managed without the modification or termination of the regimen. Few may pose a serious threat to life, requiring hospitalization or even mortality risk, compelling for modification or termination of drug regimen. Factors that may contribute to the occurrence of ADRs range from socio-demographic background to the clinical status of the patients (Edwards and Aronson, 2000). The most common ADRs on SLDs used in published literature include drug-induced hepatitis, psychotic and gastrointestinal disturbances, homeostasis fluctuations, nephrotic disorders, neuropathy, arthralgia, skin allergies, and ototoxicity (WHO, 2012; Avong et al., 2015; Falzon et al., 2017). In this context, the National Tuberculosis Program (NTP), Pakistan, and WHO recommend early identification and management of ADRs among DR-TB patients (NTP, 2017; WHO, 2019).

Programmatic management of drug-resistant tuberculosis (PMDT) was initiated in Pakistan in 2010, with 33 PMDT sites currently functional throughout the country. Few studies, to date, have reported the frequency and nature of ADRs among DR-TB patients in Pakistan (Ahmad et al., 2018; Javaid et al., 2018; Atif et al., 2022). This is the first prospective study about the frequency, management, and evaluation of risk factors for ADRs occurrence at the present site, Nishtar Medical University (NMU) Hospital, Multan, Pakistan.

## Methodology

### Study Setting

The study was conducted at the PMDT unit of NTP, Pakistan, established at the pulmonology ward, NMU, Multan, Pakistan. The pulmonology ward provides free of cost health care to DS-TB and DR-TB patients under the supervision of medical specialists (pulmonologists), pharmacists, psychologists, treatment coordinators, nurses, and other support staff. A fully equipped pathology referral laboratory at NMU provides all the necessary diagnostic services, such as smear microscopy and Xpert-MTB/RIF. The radiology and pathology department of NMU provides services to DR-TB patients on a daily basis. Drug susceptibility testing (DST) samples are referred to the national referral laboratory in Islamabad, Pakistan.

### Study Design

This was a prospective observational study of all the DR-TB confirmed patients enrolled at the study site from January 2016 to May 2017. Patients enrolled for treatment from September 2016 and onwards were followed prospectively from the baseline visit till the treatment outcome was met, while patients enrolled before September 2016 were followed retrospectively till august 2016 and then prospectively. Resistance to rifampicin is also considered multidrug resistance (MDR) and is the pre-requisite for the 18-month DR-TB treatment post sputum culture conversion with SLDs (NTP, 2014a; Ullah et al., 2016; WHO, 2019). Exclusion criteria of the study were age less than 18 years, pregnancy, and patients with intellectual disability. Participants’ marital status was categorized into married and unmarried groups as cohabitation is not in line with the cultural norms of the current study population. Baseline weight was categorized as less than 40 kg and equal to or more than 40 kg. A weight equal to or more than 40 kg has been associated with the occurrence of ADRs among DR-TB patients (Ahmad et al., 2018; Atif et al., 2022). Baseline age was categorized into three age categories (18–35, 36–50, and more than 50 years). It has been reported that age more than 50 years in Pakistan is more prone to disability or disease (Qureshi, 2017). Ethical approval was granted by the institutional ethical review board (IRB) at NMU, Multan, after the evaluation of the research study. Patients’ consent (written or oral) to the use of anonymous clinical information was obtained at the initiation of the study.

### Treatment Protocol

Patients were treated as per national and WHO guidelines. The conventional treatment therapy for DR-TB, recommended by WHO in 2011, termed a long treatment regimen, was applicable during the study period. It was comprised of at least 8-month treatment of one injectable aminoglycoside (amikacin/kanamycin/capreomycin) +levofloxacin or moxifloxacin + ethionamide + cycloserine + pyrazinamide and 12-month treatment with levofloxacin + ethionamide + cycloserine + pyrazinamide. For any patients who had a previous history of SLD use or resistance, it was recommended to add Vit B6 and para-aminosalicylic acid to the already described regimen (Falzon et al., 2011; NTP, 2014b). In 2016, WHO recommended a shorter treatment regimen (STR) for 9–11-month treatment. However, transition to STR or all oral bedaquiline based treatment regimen was yet to start at the time of initiation of the study.

Sputum samples collected from the study cohort were evaluated by direct sputum smear microscopy (Zielh-Neelson stain) and Gene Xpert/Rif (Cepheid, Sunnyvale, CA, United States). With positive smear microscopy and rapid rifampicin resistance results, patients were directed for DR-TB treatment initiation with an empirical regimen, except for those with a previous history of fluoroquinolones, as recommended by the national guidelines for DR-TB (NTP, 2014b). After the availability of sputum culture and DST results against both FLDs and SLDs, study participants were switched to an individualized regimen based on a patient-specific resistance pattern. The aim was to have at least four likely effective SLDs with a maximum recommended daily dose. Complete blood count (CBC), serum electrolytes, liver function tests (LFTs), renal function tests (RFTs), random blood glucose tests, and uric acid tests were conducted on a monthly basis. Thyroid tests, hepatitis, and HIV screening were conducted at the initiation of therapy. Visual and audiometry tests were conducted on the recommendation of the clinician for some patients and were repeated when deemed necessary on the physician’s judgment. All patients were treated free of cost on an ambulatory basis with a monthly support allowance and transportation charges.

ADRs were identified, recorded, and managed as recommended by NTP, Pakistan, criteria for PMDT sites (NTP, 2017). Patients were screened for any pre-existing symptoms before the initiation of therapy. As per PMDT protocol, the physician is required to closely monitor ADRs on a monthly basis. These may be patient-reported (subjective complaint of the sufferer), physician-observed (objective indication of disease by a health professional), or laboratory-confirmed. These were documented on the ADRs reporting form. National guidelines for PMDT were followed for the management of ADRs with medical judgement by the physician (NTP, 2014b). All patients were provided necessary education concerning drug use, compliance, possible ADRs, treatment regimen, and length of treatment. During each follow-up visit, patients were inquired about their symptoms and medication adherence. WHO operational definitions were followed regarding patient identification and diagnosis as given in Table 1 (WHO, 2013).

**TABLE 1:** Operational definitions.

Terms	Operational definitions
Mono-drug resistant TB	TB patients are non-responsive to a single anti-TB drug other than H and R in FLDs
Poly-drug resistant (PDR) TB	TB patients are non-responsive to more than one FLDs, not including H and R
Rifampicin resistant (RR) TB	TB patient non-responsive to R only
Multidrug resistant TB (MDR-TB	TB patient non-responsive to both H and R
Extensive drug-resistant TB (XDR-TB)	TB patient non-responsive to any FQs and any of the injectable SLDs (Am, Km, and Cm) in addition to H and R
Cured	DR-TB patient completes the TB treatment without any sign of therapy failure and obtains at least five consecutive negative sputum cultures in the final treatment year taken a month apart
Treatment completed	DR-TB patient completes the TB treatment with inadequate bacteriological results (less than five negative cultures in the final year of treatment) but without any sign of therapy failure
Died	DR-TB patient expires during the period of TB treatment
Treatment failed	DR-TB patient obtains two or even more positive cultures out of the five cultures during the last year of therapy or if there is a lack of improvement in therapeutic response
Defaulted	A gap of two or more consecutive months in the treatment of a DR-TB patient due to non-medical reasons
Successful outcomes	Cured or treatment completed
Unsuccessful outcomes	Died or treatment failed or defaulted
Married	Legally recognized union between people called spouses
Unmarried	Without any legally recognized union

TB, tuberculosis; FLDs, first-line anti TB drugs; SLDs, second-line anti TB drugs; H, isoniazid; R, rifampicin; FQs, fluoroquinolones; Am, amikacin; Km, kanamycin; Cm, capreomycin.

### Data Collection

A standardized form was formulated for data incorporation about patients’ demographics (age, gender, marital and residential status, and smoking), clinical history (previous TB history, length of disease, previous SLDs use, co-morbidities, lung cavitation, DST result, and resistance pattern), and baseline laboratory parameters (sputum grading and BMI) on a regular monthly basis. Patients, partially followed retrospectively, were examined from the complete patient record, including detailed laboratory parameters and ADRs reporting form with management. On each monthly visit, patients were closely monitored and evaluated for any anti-TB drug-induced ADRs by the physician and were recorded in the patients’ pharmacovigilance form purposely formulated for DR-TB therapy induced ADRs documentation. ADRs that can be verified by any laboratory value or judged by the physician based on clinical criteria, self-observed or patient-reported, the occurrence of at least one abnormal laboratory value or episode/event was considered sufficient for defining ADR (WHO, 2014a). Symptomatic management with or without regimen modification was recorded. A detailed description of the ADRs definition is provided in Table 2.

**TABLE 2:** Adverse drug reactions.

Adverse drug reactions (ADRs)	Definition
Allergic skin reaction	Any skin change characterized by pruritis, rash, acne, or photosensitivity reported by the patients and cross-examined by the physician for possible anti-TB drugs effects
Anemia	At least 1 serum hemoglobin value <13 g/dl (male) or <11.5 g/dl (female)*
Arthralgia	Joint pain reported by the patients and recorded by the physician with or without arthritis
Body pain and headache	As reported by the patients
Dyspnoea	Any difficulty in breathing as reported by the patients and evaluated by the physician
Gastrointestinal disturbances	Any case of gastritis, nausea, vomiting, abdominal pain, or diarrhea reported by the patients or documented by the physician
Gynecomastia	Consistent and painful enlargement of breast documented by the physician for possible anti-TB drug effects
Hemoptysis	Spitting of blood with cough as reported by the patients and documented by the physician
Hepatotoxicity	In the absence of symptoms, at least 1 elevated serum value of transaminase or bilirubin five times higher than the upper normal value or three times higher in case of symptoms
Hypothyroidism	At least 1 increased serum TSH value >10 IU/ml*
Hypokalemia	1 serum potassium value <3 mmol/L*
Hyperuricemia	At least 1 serum increased value of uric acid >9 mg/dl*
Nephrotoxicity	At least 1 serum creatinine value >130 μmol/L*
Ototoxicity	Hearing loss or tinnitus as reported by the patients and confirmed by audiometry or clinician examination
Peripheral neuropathy	Symptoms linked with numbness or burning sensation of extremities as reported by the patients and evaluated by the physician
Psychiatric disturbances	Any cases of depression or psychosis evaluated by a psychologist or psychiatrist
Sleep disturbances	As reported by the patients and documented by the physician
Swelling	Evaluated by the physician by physical examination
Vertigo and dizziness	As reported by the patients and evaluated by the physician
Visual disturbances	DIFFICULTY in vision as reported by the patients

Normal Ranges: Alanine aminotransferases (ALT) = up to 41 U/L; Bilirubin total = Up to 1.2 mg/dl; serum creatinine = 0.74–1.35 mg/dl; Hemoglobin (Hb) Male (13–18) g/dl Female (11.5–16.5) g/dl.

### Statistical Analysis

Clinical data obtained were analyzed using SPSS 26, IBM Corp. for Windows™. Descriptive analysis was performed to describe study enrolled participants. For a continuous variable, mean and standard deviation were used, while frequencies and percentages were used for categorical variables. Regression analysis was used to evaluate the relationship between study participants and the occurrence of ADRs (dependent variable). Variables having *p* < 0.05 in univariate analysis were considered for multivariate binary logistic regression analysis to establish a possible link between the occurrence of ADRs and any affecting variable. Statistical significance was set to be *p* < 0.05.

## Results

### Demographic Data

As per sample size calculation, 246 patients were calculated based on the DR-TB incidence rate in Pakistan in 2016. After including the 20% dropout criteria, the sample size was calculated to be 308 (USM, 2017). Excluded patients included five pregnant women at the start of the study, 31 patients less than 18 years old, and one patient with an intellectual disability, thus making the total sum of 37 participants. The remaining 271 patients, who had met their final treatment outcome, whether successful (cured and completed) or unsuccessful (died, failed, or defaulted), were included in the study.

### Patients’ Characteristics and Frequency of Adverse Drug Reactions

Males (*n* = 139) and females (*n* = 132) were nearly in an equal proportion in the study. The mean age of all the participants was 36.75 (SD = 15.69) years. The mean baseline body weight was 45.44 (SD = 11.61) kg. Non-smoker patients (*n* = 248, 88.6%) constituted the major proportion of the study. Most of the participants were married (*n* = 195, 72%). Rural and urban participants were 131 (48.3%) and 140 (51.7%), respectively. The illness duration for nearly half of the study participants (53.5%) before DR-TB diagnosis was between 6–12 months. Most of the patients weighed less than 40 Kg. Half of the patients (56.8%) had bilateral lung cavitation at the baseline visit. None of the patients had positive HIV status. DR-TB cases, in the current study, used an average of 5 (4–9) most likely effective drugs in the intensive phase of treatment, while an average of 6.38 (5–12) anti-TB drugs throughout the treatment. The median length of injectable administration was nine months (range 4–14), and the median length of DR-TB treatment was 20 months (range 1–32). Successful treatment outcome (cured and completed) was noted for 187 (69%), while unsuccessful treatment outcome was observed for 48 (17.7%) died, 34 (12.5%) defaulted, and 2 (0.7%) treatment failure. A detailed description of the patient’s socio-demographic characteristics and clinical history has been provided in Table 3.

**TABLE 3:** Patients’ characteristics and frequency of ADRs.

Characteristics		Adverse drug reactions *n* (%)	
	*n* (%)	Yes (*n* = 220)	No (*n* = 51)
Gender		221 (81.2)	51 (18.8)
Male	139 (51.3)	111 (79.9)	28 (20.1)
Female	132 (48.7)	109 (82.6)	23 (17.4)
Age (years) (Mean ± SD = 36.75 ± 15.69)			
18–35 years	139 (51.3)	122 (87.8)	17 (12.2)
36–50 years	80 (29.5)	62 (77.5)	18 (22.5)
>50 years	52 (19.2)	36 (69.2)	16 (30.8)
Patient weight at baseline (kg) (Mean ± SD = 45.44 ± 11.61)			
<40 kg	188 (69.4)	153 (81.4)	35 (18.6)
≥40 kg	83 (30.6)	67 (80.7)	16 (19.3)
Marital Status			
Unmarried	76 (28)	70 (92.1)	6 (7.9)
Married	195 (72)	150 (76.9)	45 (23.1)
Residence			
Rural	131 (48.3)	108 (82.4)	23 (17.6)
Urban	140 (51.7)	112 (80)	28 (20)
Smoking history			
Non-smoker	240 (88.6)	194 (80.8)	46 (19.2)
Active and ex-smoker	31 (11.4)	25 (80.6)	5 (19.4)
Duration of illness prior to DR-TB diagnosis			
Less than 6 months	74 (27.3)	64 (86.5)	10 (13.5)
6–12 months	145 (53.5)	117 (80.7)	28 (19.3)
1–2 years	37 (13.7)	27 (73)	10 (27)
More than 2 years	15 (5.5)	12 (80)	3 (20)
Treatment category			
New	38 ()14	32 (84.2)	6 (15.8)
Relapse	6 (2.2)	3 (50)	3 (50)
Treatment after failure	198 (73.1)	161 (81.3)	37 (18.7)
Treatment after loss to follow-up	26 (9.6)	22 (84.6)	4 (15.4)
Others	3 (1.1)	2 (66.7)	1 (33.3)
Previous TB treatment			
Yes	233 (86.3)	188 (80.7)	45 (19.3)
No	37 (13.7)	31 (83.8)	6 (16.2)
Resistance to All FLDs			
Yes	14 (5.2)	12 (85.7)	2 (14.3)
No	257 (94.8)	208 (80.9)	49 (19.1)
Previous use of SLDs			
Yes	17 (6.5)	12 (70.6)	5 (29.4)
No	244 (93.5)	198 (81.1)	46 (18.9)
Resistance to any SLDs			
Yes	71 (26.2)	57 (80.3)	14 (19.7)
No	200 (73.8)	163 (81.5)	37 (18.5)
Co-morbidity			
Yes	45 (16.6)	38 (84.4)	7 (15.6)
No	226 (83.4)	182 (80.5)	44 (19.5)
Patients’ family TB history/status			
No TB	251 (92.6)	206 (82.1)	45 (17.9)
DS-TB	14 (5.2)	11 (78.6)	3 (21.4)
DR-TB	6 (2.2)	3 (50)	3 (50)
Hemoglobin level at baseline			
Normal	64 (27)	56 (87.5)	8 (12.5)
Less than normal	173 (73)	150 (86.7)	23 (13.3)
Baseline sputum grading			
Negative	22 (8.1)	18 (81.8)	4 (18.2)
Scanty^a^, +1^b^	134 (49.4)	107 (79.9)	27 (20.1)
+2^c^, +3^d^	115 (42.4)	95 (82.6)	20 (17.4)
Lung cavitation at baseline			
No cavitation	50 (18.5)	30 (60)	20 (40)
Unilateral cavitation	67 (24.7)	62 (92.5)	5 (7.5)
Bilateral cavitation	154 (56.8)	128 (83.1)	26 (16.9)
Body mass index (BMI)			
Underweight	41 (15.1)	31 (75.6)	10 (24.4)
Normal	77 (28.4)	66 (85.7)	11 (14.3)
Overweight	153 (56.5)	123 (80.4)	30 (19.6)
Patient resistance category			
RR only	134 (49.4)	110 (82.1)	24 (17.9)
PDR	1 (0.4)	1 (100)	0
MDR	128 (47.2)	104 (81.3)	24 (18.8)
XDR	8 (3)	5 (62.5)	3 (37.5)
Treatment outcome category			
Cured	187 (69)	182 (82.7)	5 (9.8)
Died	48 (17.7)	29 (60.4)	19 (39.6)
Failed	2 (0.7)	2 (100)	0
Loss to follow-up (defaulted)	34 (12.5)	7 (20.6)	27 (79.4)

FLDs, first-line anti-TB drugs; SLDs, second-line anti-TB drugs; DS-TB, drug-susceptible TB; DR-TB, drug-resistant TB; kg, kilogram; SD, standard deviation.

a 1–9 Acid-fast bacilli (AFB)/100 high power field (HPF).

b 10–99 AFB/100HPF.

c 1–9 AFB/HPF.

d >9 AFB/HPF.

### Patients’ Treatment Regimen

Most of the patients were administered at least four or more likely effective drugs in the intensive phase. Patients were kept on empirical therapy until the availability of DST results. After the availability of DST results, an individualized regimen was started. The detail of drugs used, along with daily doses and frequency of patients using each drug, has been described in Table 4.

**TABLE 4:** Drugs used along with daily dose and frequency of patients receiving each drug.

Group	Drugs	Recommended dose (mg/kg of body weight)	Frequency of patients receiving each drug *n* (%)
Group 1 FLDs	H	16–20 once daily (Max dose 1,500 mg)	0
	E	25 once daily (Max dose 2,000 mg)	73 (26.9)
	Z	30 to 40	269 (99.3)
Group 2 injectable anti-TB drugs	Am	15 to 20	249 (91.9)
	Cm	15 to 20	35 (12.9)
	Km	15 to 20	0
	S	12–18 once daily (Max dose 1,000 mg)	1 (0.4)
Group 3 fluoroquinolones	Lfx	7.5 to 10	241 (88.9)
	Mfx	7.5 to 10	33 (12.2)
Group 4 oral bacteriostatic SLDs	Eto	15 to 20	271 (100)
	Pto	15 to 20	0
	Cs	15 to 20	271 (100)
	PAS	150 (Max dose 8–12 g)	236 (87.1)
	Cfz	100 mg once daily	19 (7)
	Lzd	600 my once daily	19 (7)
	Bdq	400 mg once daily for 2 weeks and then 200 mg three times per week	1 (0.4)
Group 5 anti-TB drugs with limited data available on the efficacy	Amx/Clv	1,500/375 mg daily	12 (4.4)
	Clr	1,000 mg once daily	8 (3)

H, isoniazid; E, ethambutol; Z, pyrazinamide; Cs, cycloserine; Eto, ethionamide; PAS, para-amino salicylic acid; Am, amikacin; Cm, capreomycin; Km, kanamycin; S, streptomycin; Lfx, levofloxacin; Mfx, moxifloxacin; Pto, prothionamide; Cfz, clofazimine; Bdq, bedaquiline; Amx, Amoxicillin; Clv, Clavolonate; Lzd, linezolid; Clr, clarithromycin.

### Resistance Pattern Among Study Patients

Among all the patients, RR-TB, MDR-TB, XDR-TB, and PDR-TB patients constituted 134 (49.45%), 128 (47.23%), 8 (2.95%), and 1 (0.3%), respectively.

### Types of ADRs, Their Frequency, and Management

Among all the patients who were enrolled in the study, a total of 718 ADRs were observed. The occurrence and frequency of various ADRs were reported during the treatment. Gastrointestinal disturbances (66.7%), nervous system disorders (59.4%), and electrolyte disturbances (55.7%) remained the highest reported ADRs during therapy. These ADRs were followed by arthralgia (49.1%), ototoxicity (24%), pruritic reactions/rash (12.9%), dyspnoea (12.5%), and tinnitus (8.8%). A small number of patients reported some less frequent ADRs such as nephrotoxic, peripheral neuropathy, gynecomastia, menstrual cycle irregularities, memory loss, haemoptysis, visual disturbances, anaemia, anorexia, dizziness/vertigo, and photosensitivity. Life-threatening ADRs were not common. Arthralgia was associated with hyperuricaemia in only one patient out of 137. The majority of the ADRs reported were during the intensive phase and were managed with ancillary or symptomatic treatment. Identification of suspected drugs, frequency, and management of ADRs among DR-TB patients has been described in Table 5.

**TABLE 5:** Identification of suspected drugs, frequency, and management of ADRs among DR-TB patients (*n* = 271).

Adverse drug reaction	Suspected drugs	ADR frequency (%)	Action taken	Modified RR/MDR-TB regiment	Type of Action taken		
					Dose reduction	Temporary withdrawal	Permanent withdrawal
Gastrointestinal Disturbances		181 (66.7)					
Gastrointestinal upset	H, PAS, Cs, Eto	146 (53.8)	Counselled and reassured. Patients were prescribed *PPI* along with *prokinetics* drugs. Medication was modified for one patient and was replaced with *Lzd*	PAS (1)			PAS (1)
Nausea and Vomiting	PAS	29 (10.7)	Counselled and reassured. All patients were prescribed d*omperidone* along with *prokinetics* drugs. PAS dose was reduced in one patient		PAS (1)		
Diarrhea	PAS, H	4 (1.47)	Patients were prescribed *diosmectite* powder				
Hiccups	—	1 (0.36)	The patient was prescribed *Baclofen* along with *PPI*				—
Oral Ulcer	—	1 (0.36)	The patient was prescribed *Lignocaine* gel and advised for use of mouthwash				
Nervous System Disorders	Cs	161 (59.4)		—	—	—	—
Depression	Cs	75 (27.6)	Counselled and prescribed *SSRI* antidepressants. The dose of *Vit B6* was also increased. The offending agent was withheld temporarily in one patient and dose was reduced in another patient	Cs (2)	Cs (1)	Cs (1)	—
Sleep disturbances	Cs	48 (17.7)	All patients were counselled and prescribed *Benzodiazepines*	None	—	—	—
Psychosis	Cs	27 (10)	All the patients were prescribed antipsychotic medication after referral to the psychiatry ward. 27 patients were prescribed *risperidon*e. The offending drug was temporarily and permanently stopped in two and one patient, respectively, and dose was reduced in one patient	Cs (5)	Cs (2)	Cs (2)	Cs (1)
Aggression	Cs	5 (1.84)	Counselled and referred to the psychiatry ward. All patients were prescribed *Fluphenazine HCl* and *nortriptyline*	None	—	—	—
Visual disturbances	—	4 (1.47)	Patients were counselled and were referred to an ophthalmologist. *Vit B6* dose was increased in all patients	None			
Memory loss	Cs	2 (0.7)	The patients were prescribed a higher dose of *Vit B6*	None	—	—	—
Electrolyte disturbances	Am	151 (55.7)	Patients were monitored and two patients were advised for potassium-rich food and a *potassium* supplement was added to two patients’ treatment regimen due to Hypokalemia	None			
Arthralgia/Hyperuricemia	Z, Cs, H	137 (49.1)	Patients were prescribed *NSAIDs* for symptomatic relief. One patient was prescribed *Allopurinol* and dose of Z was reduced in another patient	Z (1)	Z (1)		
Ototoxicity	Am, S	65 (24)	Counselled. Am was replaced to Cm in 33 patients and dose was reduced in nine patients while use was withheld temporarily and permanently in one and three patients, respectively. Nine patients were kept without management as they had completed their injectable treatment and this ADR was subsided	Am (45) S (1)	Am (9)	Am (1)	Am (3)
Pruritis/Rash/Acne	Am	35 (12.9)	Counselling was provided to patients. *Antihistamine* and *hydrocortisone* therapy was prescribed along with the use of r	Skin emollients. *Fusidic acid* was prescribed for one patient. Two patients were prescribed *anti-acne* therapy	None		
Dyspnoea		34 (12.5)	All patients were counselled and prescribed a *bronchodilator*	None			
Body Pain and Headache		27 (10)	All patients were counselled and prescribed *NSAIDs*	None			
Tinnitus	Am, Cm, Eto	24 (8.8)	All patients were counselled and prescribed *Betahistine*. The Injection was stopped temporarily in one patient and advised on an alternate day in another patient	Cm (2)	Cm (1)	Cm (1)	—
Peripheral neuropathy	Cs	14 (5.2)	All patients were counselled and prescribed *duloxetine* and *vit B6* dose was increased	None			
Anorexia	—	7 (2.58)	All patients were prescribed *appetizers* and *multivitamins*	None			
Hypothyroidism	PAS	5 (1.84)	All were counselled and prescribed *thyroxine*. Use of the offending agent was stopped in one patient and dose was reduced in another patient	PAS (2)	PAS (1)		PAS (1)
Dizziness	Z, Cs	4 (1.47)	Patients were counselled. The dose of *Vit B6* was increased in all patients. Z was advised to be taken on an alternate day in one patient. one patient was prescribed *Prochlorperazine*, and another patient was prescribed *Betahistine*	Z (1)	Z (1)		
Hemoptysis		3 (1.1)	Patients were counselled. All three patients were prescribed *Tranexamic acid*	None			
Anemia	PAS	2 (0.7)	Patients were counselled. One patient was prescribed for iron supplement and one patient was advised for blood transfusion along with the temporary withdrawal of the offending agents	PAS (2)		PAS (2)	
Nephrotoxicity	Cm	2 (0.7)	Patients were counselled and prescribed prednisolone along with the removal of the offending agent permanently in one patient and replaced with *Lzd* in another patient	Cm (2)			Cm (2)
Palpitations		2 (0.7)	Counselling was provided to the patients. *Beta-blockers* were prescribed	None			
Gynecomastia		2 (0.7)	*NSAIDs* were added to patients’ treatment along with counselling	None			
Menstrual irregularities	Eto	1 (0.36)	The patient was counselled and referred to a gynecologist	None			
Photosensitivity	Eto	1 (0.36)	The patient was prescribed a higher dose of *Vit B6*	None			
Swelling		1 (0.36)	The patient was counselled and prescribed a *diuretic*	None			

H, isoniazid; Cs, cycloserine; Eto, ethionamide; PAS, para-amino salicylic acid; Z, pyrazinamide; Am, amikacin; Cm, capreomycin; S, streptomycin; NSAID, non-steroidal anti-inflammatory drugs; Lzd, linezolid; vit, vitamin.

### Factors Associated With the Occurrence of Adverse Drug Reactions

The variables that emerged with possible association of occurrence of ADRs included age, being unmarried (*p*-value = 0.006) OR 3.5; 95% CI (1.426–8.590), hemoglobin level at baseline less than normal (*p*-value = 0.028) OR 2.023; 95% CI (1.079–3.793), and lung cavitation at baseline (*p*-value = 0.000) with OR 4.086; 95% CI (2.067–8.076) as mentioned in Table 6. When multivariate binary logistic regression was applied as given in Table 7, lung cavitation at baseline (*p*-value = 0.001) OR 3.419 (1.694–6.902) emerged as the only variable associated with the occurrence of ADRs.

**TABLE 6:** Univariate analysis of risk factors associated with adverse drug reactions.

Variable	Odds ratio (95% CI)	*p*-value
Gender		
Female	Referent	0.567
Male	0.876 (0.454–1.542)	
Age (years) (Mean ± SD = 36.75 ± 15.69)		
18–35 years	Referent	**0.011**
36–50 years	0.48 (0.231–0.996)	**0.049**
>50 years	0.314 (0.144–0.682)	**0.003**
Patient weight at baseline (kg) (Mean ± SD = 45.44 ± 11.61)		
<40 kg	Referent	0.898
≥40 kg	0.958 (0.496–1.849)	—
Marital Status		
Married	Referent	**0.006**
Unmarried	3.5 (1.426–8.590)	
Residence		
Rural	Referent	0.607
Urban	0.852 (0.462–1.570)	
Smoking history		
Non-smoker	Referent	0.742
Active and ex-smoker	1.186 (0.431–3.263)	
Duration of illness prior to DR-TB diagnosis		
Less than 6 months	Referent	0.397
6–12 months	0.653 (0.298–1.430)	0.286
1–2 years	0.422 (0.158–1.130)	0.086
More than 2 years	0.625 (0.150–2.612)	0.519
Treatment category		
New	Referent	0.403
Relapse	0.188 (0.03–1.16)	0.072
Treatment after failure	0.816 (0.318–2.093)	0.672
Treatment after loss to follow up	1.031 (0.26–4.086)	0.965
Others	0.375 (0.029–4.821)	0.452
Previous TB treatment		
No	Referent	0.655
Yes	0.809 (0.318–2.055)	
Resistance to All FLDs		
No	Referent	0.657
Yes	1.413 (0.306–6.521)	
Previous use of SLDs		
No	Referent	0.294
Yes	0.558 (0.187–1.661)	
Resistance to any SLDs		
No	Referent	0.822
Yes	0.924 (0.466–1.833)	
Co-morbidity		
No	Referent	0.541
Yes	1.312 (0.549–3.135)	
Patients’ family TB history/status		
No TB	Referent	0.183
DS-TB	0.801 (0.215–2.989)	0.741
DR-TB	0.218 (0.043–1.118)	0.068
Hemoglobin level at baseline		
Normal	Referent	**0.028**
Less than normal	2.023 (1.079–3.793)	
Baseline sputum grading		
Negative	Referent	0.855
Scanty^a^, +1^b^	0.881 (0.275–2.817)	0.83
+2^c^, +3^d^	1.056 (0.322–3.455)	0.929
Lung cavitation at baseline		
No cavitation	Referent	
Cavitation	4.086 (2.067–8.076)	**0.00**
Body mass index (BMI)		
Under weight	Referent	0.386
Normal	1.935 (0.743–5.039)	0.176
Overweight	1.323 (0.584–2.994)	0.502

SLDs, second line anti-TB drugs; DS-TB, drug susceptible TB; DR-TB, drug resistant TB; kg, kilogram; SD, standard deviation.

a 1–9 Acid-fast bacilli (AFB)/100 high power field (HPF).

b 10–99 AFB/100HPF.

c 1–9 AFB/HPF.

d >9 AFB/HPF; FLDs, first-line anti-TB drugs.

Bold values means that p‐value is significant.

**TABLE 7:** Multivariate analysis of risk factors for occurrence of adverse drug reactions.

Variable	B	S.E.	OR (95% CI)	*p*-value
Age (18–35) years			Referent	0.306
36–50 years	−0.067	0.443	0.935 (0.392–2.23)	0.88
>50) years	−0.618	0.457	0.539 (0.22–1.32)	0.176
Unmarried	0.892	0.542	2.441 (0.844–7.061)	0.1
Baseline hemoglobin level	0.499	0.342	1.648 (0.843–3.222)	0.144
Baseline lung cavitation	1.229	0.358	3.419 (1.694–6.902)	**0.001**

Bold values means that p‐value is significant.

## Discussion

The emergence of drug resistance in TB has posed a global threat due to its infectious nature and is considered fatal. The complex combination of drug therapy with associated ADRs has made it quite difficult to efficiently manage patients. In the present study, 220 (81%) patients experienced ADRs. However, the majority of the ADRs were resolved with collective efforts of physician-led interventions combined with psychologist and family support, and none of the ADRs progressed to any permanent termination of therapy among study participants.

The frequency of ADRs in the current study was found to be in line with already published studies from Russia (73.3%) (Shin et al., 2007), Pakistan (72%) (Ahmad et al., 2018), Turkey (69%) (Törün et al., 2005), and India (57.6%) (Dela et al., 2017). A higher frequency was reported in a study where nearly all of the patients (99%) experienced at least one ADR (Ganiyu et al., 2021). The present study was supported by some other studies conducted in Latvia (79%) (Bloss et al., 2010), China (90.7%) (Zhang et al., 2017), Indonesia (70%) (Nilamsari et al., 2021), and Italy (89%) (Gualano et al., 2019). Likewise, three studies reported from Pakistan also reported the occurrence of ADRs ranging from 63% to 77% (Ahmad et al., 2018; Javaid et al., 2018; Atif, 2021). Contrary to already mentioned, studies from South Africa (38.9%), Ethiopia (51%) (Merid et al., 2019), and India (47%), where lower ADRs frequency was reported. The varying differences in the frequency of ADRs in reported studies may be due to differences in attitudes towards therapy, such as lack of treatment adherence, default rate, differences in opinions of patients and physicians with respect to ADRs reporting, ability to detect, drug use pattern, differences in a support program, early assessment, and management of ADRs (Li et al., 2014; Akshata et al., 2015a; Hoa et al., 2015; Kelly et al., 2016; Dela et al., 2017; Zhang et al., 2017; Prasad et al., 2019). Nutritional practices, geographic location, age, patient awareness, and nature of co-morbidity with DR-TB are some of the patient-relevant factors (Zhang et al., 2017; Merid et al., 2019). A lack of harmony regarding the ADRs reporting among patients and physicians was reported in one of the studies. Patients had reported more ADRs than those documented by the clinicians (Kelly et al., 2016). This reflected the perception differences about ADRs between physicians and patients. Another study reported the lack of provision of the required information about the regimen (Atif et al., 2016). Inadequate knowledge about drugs and drug-induced ADRs leads to patients’ erroneous ADRs reporting (Partnership, 2015). The complex nature of the regimen with the presence of co-morbidity leads to a higher risk of ADRs occurrence, as widely reported in published literature (Furin et al., 2001; Mouton et al., 2016; Schaaf et al., 2016; Merid et al., 2019).

In our study, gastrointestinal disturbances [*n* = 181 (66.7%)] appeared as one of the most reported ADRs. Our findings are consistent with the studies reporting prevalence ranging from 10 to 100% in various groups undergoing DR-TB treatment (Hoa et al., 2015; Ahmad et al., 2018; Furin et al., 2001; Lakhani et al., 2019; Ganiyu et al., 2021). These disturbances were reported with an incident rate of 32%, according to a meta-analysis of 28 studies comprising approximately 4000 DR-TB patients (Wu et al., 2016). Subgroup disturbances included gastritis (53%), nausea and vomiting (10.7%), diarrhea (1.47%), and a few cases of oral ulcers and hiccups. Similar findings with higher frequency were reported in studies reported from Pakistan (42%) (Ahmad et al., 2018), Ethiopia (59%) (Bezu et al., 2014), India (71%) (Akshata et al., 2015a), and Russia (75%) (Shin et al., 2007). Among all the patients who reported gastrointestinal disturbances, regimen modification was considered in only two patients. The suspected drug was replaced in one patient, and the dose of the suspected drug was reduced in the other. All other patients managed these ADRs with ancillary drugs comprising antiemetics, prokinetics, and proton pump inhibitors. Similar management was reported in another study conducted among MDR-TB patients in Pakistan (Ahmad et al., 2018). Even though gastrointestinal disturbances occur more frequently as compared to any other ADR, most patients need symptomatic therapy due to the mild to moderate nature of severity, thus avoiding termination of the causative agent (Nathanson et al., 2004; Carroll et al., 2012; Furin et al., 2001; Prasad et al., 2019).

Nervous system disorders were reported in 59.4% of the study cohort. Among these, depression (27.6%), sleep disturbances (17.7%), psychosis (10%), aggression (1.84%), visual disturbances (1.47%), and memory loss (0.7%) were reported. These findings are supported by similar findings reported in Pakistan (29%) (Ahmad et al., 2018) and Egypt (26.5%) (Elmahallawy et al., 2012). The psychiatric disorder prevalence was found to be in a range of 4% to 36% in individual studies among DR-TB patients (Furin et al., 2001; Hoa et al., 2015). Anti-TB drugs suspected of psychiatric complications include isoniazid, fluoroquinolones, ethionamide, and cycloserine (Carroll et al., 2012; WHO, 2014b; Gupta et al., 2020). Apart from ADRs, societal stigma attached to the disease, the length of therapy, financial problems, and any previous therapy experiences also severely affect patients (Tandon et al., 1980; Barnhoorn and Adriaanse, 1992; Kiecolt-Glaser and Glaser, 2002; Rajeswari et al., 2005; Furin et al., 2014). Reported studies have mentioned the usefulness of discontinuation of cycloserine in some patients (Prasad et al., 2016; Dela et al., 2017; Ahmad et al., 2018). The psychological and psychiatric disturbances of lesser severity can be managed by a specialized healthcare professional, social support, or by prescribing anti-depressants to DR-TB patients. In severe cases, the offending drug can be terminated or replaced with other alternatives (Gupta et al., 2020). In our research, cycloserine was suspected to be the main culprit drug. It was temporarily removed from therapy in two patients, whereas it was permanently removed for one patient diagnosed with psychosis. The dose was also reduced for one patient. The rest of the patients were managed with counselling and prescribing anti-psychotic drugs, which led to the resolution of psychosis in all patients. All patients (75) diagnosed with depression were counselled by a psychologist and were prescribed SSRIs. Cycloserine was withheld temporarily in one patient while the dose was reduced in another depression patient. One of the meta-analyses suggested for removal of cycloserine without compromising the treatment outcome among DR-TB patients and recommended clofazimine, fluoroquinolones, or bedaquiline use if the DST results do not recommend otherwise (Lan et al., 2020).

Other frequently reported nervous system disorder was difficulty in sleeping. Around 17.7% of patients reported sleep disturbances. Similar findings were reported from a study held in Egypt (22.5%), while another study from Bangladesh reported 44% sleep disturbances (Ibrahim et al., 2017; Masuma et al., 2018). The differences in various studies may be attributed to the patient-reported versus physician-documented ADRs as reported in a study from the United States, where insomnia was the most common reported ADR (67%) by the patients, while clinicians documented only 2% of the sleep disturbances (Kelly et al., 2016). All patients were counselled and reassured for therapy continuation. Benzodiazepines were prescribed for sleep disturbances without the need for treatment modification.

Despite the higher frequency of electrolyte disturbances (55%), hypokalemia was diagnosed in only two patients who were advised for potassium-rich food and the addition of potassium supplements in their treatment regimen without causing any treatment modification. The low incidence of hypokalemia may be attributed to the aggressive and efficient management of DR-TB therapy with continuous monitoring.

Arthralgia was reported in 49% of the study participants with varying degrees of severity during anti-TB therapy (Wu et al., 2016; Sineke et al., 2019; Gupta et al., 2020; Tornheim et al., 2021). Similar findings of higher frequency were reported in studies conducted in Russia (47%) (Shin et al., 2007) and China (56.4%) (Zhang et al., 2017), whereas a lower incidence was reported in studies from Namibia (26%) (Sagwa et al., 2013) and Ethiopia (34%) (Bezu et al., 2014). Joint pain was among the most reported ADR while evaluating the health-related quality of life in the DR-TB cohort (Sineke et al., 2019). Bedaquiline, fluoroquinolones, streptomycin, ethambutol, and pyrazinamide are thought to be involved in arthralgia by causing hyperuricemia (Gerdan et al., 2013; Wu et al., 2016; Gupta et al., 2020; Lan et al., 2020). Joint pain developed during therapy subsides with non-steroidal anti-inflammatory drugs (NSAIDs), uric acid lowering agents, or taking sufficient rest (Gupta et al., 2020). In the current study, all the patients were prescribed *NSAIDs* for symptomatic relief. One patient was prescribed *Allopurinol*, and the dose of pyrazinamide was reduced in another patient.

Hearing loss was reported in 24% of the patients in the present study, which is in line with the already reported 28.3% incidence in a cohort of 12793 DR-TB patients. The occurrence of hearing loss was highest among patients using amikacin and lowest in patients using capreomycin (Wrohan et al., 2021). Similar findings were reported in studies conducted elsewhere (Wu et al., 2016). Higher dose per body weight on a monthly basis [adjusted odds ratio (aOR)] 1.15, 95% CI 1.04–1.28 and longer duration of amikacin (aOR1.98, CI 1.04–2.12) use are associated with the development of ototoxicity (Modongo et al., 2014). Amikacin was the causative agent for this disability of mild to moderate severity in the current study. Amikacin was replaced with capreomycin in thirty-three patients, and the dose was reduced in nine patients. Use was withheld temporarily and permanently in one and three patients, respectively. Nine patients were kept without management because they had completed their injectable treatment and the ADR subsided gradually. None of the patients developed any permanent loss.

Pruritis, rash, or acne was reported in 35 (12.9%) patients. Similar findings have been reported with varying frequencies ranging from 45% to 2.64% (Bezu et al., 2014; Akshata et al., 2015b; Rathod et al., 2015; Nagpal et al., 2018). Pyrazinamide and amikacin were suspected as the causative agents. Antihistamine and hydrocortisone therapy was prescribed with the use of skin emollients. Fusidic acid was prescribed for one patient. Two patients were prescribed anti-acne therapy.

Dyspnea was observed in 12.5% of the patient with mild to moderate severity. All patients were counselled and were prescribed bronchodilators. Tinnitus was reported by 9% of the patients. All patients were prescribed betahistine. Capreomycin was stopped temporarily in one patient and was advised to be taken on alternate days in another patient. Anemia was reported in two patients; one patient was prescribed an iron supplement, and the other was advised for blood transfusion along with the temporary withdrawal of para-amino salicylic acid.

Peripheral neuropathy was reported in a relatively small number (5.2%) of patients that adversely affects the daily quality of life. Higher frequency was reported in studies in Bangladesh (28%) (Masuma et al., 2018), Russia (13%) (Shin et al., 2003), and India (18.75%) (Tiwari et al., 2015). Comparable findings to the current study were reported from Pakistan (2.2%) (Ahmad et al., 2018). Anti-TB drugs such as linezolid, cycloserine, isoniazid, fluoroquinolones, SLDs, ethambutol, and ethionamide are blamed for peripheral neuropathy (Gupta et al., 2020). These drugs interfere with pyridoxine metabolism by various mechanisms (Ebadi et al., 1982; Cohen, 2001). In the present study, only cycloserine was suspected of causing neuropathy. Pyridoxine has been prescribed for the management of peripheral neuropathy along with a tricyclic antidepressant, usually amitriptyline. In cases of severity, one or more offending drugs were terminated or temporarily removed (Shin et al., 2007; Brust et al., 2013; Gupta et al., 2020). All patients were prescribed duloxetine for nerve pain and fibromyalgia. The dose of vitamin B6 was increased, and no modifications were made to the regimen.

Nephrotoxicity was not common in our study, with only 2 (0.7%) patients having toxic effects of anti-TB drugs as mentioned in the literature (Shin et al., 2007; Bezu et al., 2014; Furin et al., 2001; Ahmad et al., 2018). A study from Bangladesh reported an increased creatinine level among 3% of the DR-TB patients (Masuma et al., 2018). The most common nephrotoxic drug used for DR-TB treatment was capreomycin, followed by kanamycin and amikacin (Shibeshi et al., 2019). Patients were counselled and prescribed prednisolone along with the permanent removal of the offending agent in one patient and replacement with linezolid in another patient.

Hypothyroidism was also a less frequent observation (1.84%), and patients were counselled and were prescribed thyroxine. The use of para-amino salicylic acid was stopped in one patient, and the dose was reduced in another patient. Gynecomastia, menstrual cycle irregularities, haemoptysis, memory loss, visual disturbances, anorexia, dizziness/vertigo, and photosensitivity were some of the lower frequency ADRs in this cohort. Encouragingly, no case of hepatotoxicity was reported in our cohort.

In our study, after statistical analysis, lung cavitation at the baseline was linked to the higher probability of ADRs among DR-TB patients. Patients with cavitation are at higher odds of developing ADRs than those without pulmonary cavitation, which is consistent with the findings already reported among DR-TB patients in Pakistan (Javaid et al., 2018). Identification of predictors for factors that may contribute to the probability of occurrence of ADRs can help to develop individual and focused monitoring plans, which may result in enhanced patient compliance and better disease management. The present study also found that successful outcomes had a significant correlation with the occurrence of ADRs. It is considered that patients may terminate therapy due to ADRs and sometimes may miss the dose of the suspected drug without informing the physician. The analysis of predictors for successful treatment outcomes was not in the scope of this study.

One of the limitations of our study is the absence of documentation of the severity of ADRs, which would have helped to assess ADR severity impact on the treatment outcome. Another limitation may be the absence of medication records for co-morbidities in patients having any co-morbidity. One of the key areas of ADRs reporting is discord between physicians and patients about certain ADRs, such as nausea, vomiting, dizziness, body pain, and headache (Kelly et al., 2016). The 69% treatment outcome of this study is quite below the WHO criterion of 75%; therefore, it is recommended to put all possible efforts into better and enhanced management of treatment plans, especially for loss to follow-up patients. Although this was the first prospective study at the current study site, it is emphasized to have multicenter prospective studies for better assessment of the treatment efficacy. Regular clinical monitoring and quality laboratory analysis along with multidisciplinary approaches are needed to avoid the unsuccessful outcomes.

## Conclusion

ADRs were highly prevalent in the current study but most of them were managed with pharmacological, non-pharmacological, and psychological approaches with limited modifications in treatment plan. The higher frequency of amikacin-related temporary hearing loss leading to the treatment modification among patients is of concern. This necessitates regular audiometry to avoid any permanent hearing loss. Fewer cases of replacement of suspected drugs with alternatives, without adversely impacting the treatment outcome were observed. Hence, it highlights the importance of individualized and continuous monitoring throughout the therapy.

## Data Availability

The raw data supporting the conclusions of this article will be made available by the authors, without undue reservation.
